# Transcatheter Aortic Valve Replacement in Patients With Quadricuspid Aortic Valve: A Case Series and Systematic Review

**DOI:** 10.1155/crp/7815279

**Published:** 2025-02-05

**Authors:** Wenjing Sheng, Dao Zhou, Hanyi Dai, Rongrong Zheng, Ailifeire Aihemaiti, Xianbao Liu

**Affiliations:** ^1^Department of Cardiology, Second Affiliated Hospital Zhejiang University School of Medicine, Hangzhou, China; ^2^State Key Laboratory of Transvascular Implantation Devices, Hangzhou 310009, China; ^3^Binjiang Institute of Zhejiang University, Hangzhou, Zhejiang 310052, China

**Keywords:** long-term prognosis, quadricuspid aortic valve, technical success, transcatheter aortic valve replacement

## Abstract

**Background:** Quadricuspid aortic valve (QAV) is a rare congenital cardiac anomaly associated with symptomatic aortic regurgitation (AR) or aortic stenosis (AS). Transcatheter aortic valve replacement (TAVR) for QAV remains uncertain.

**Methods:** We retrospectively reviewed prospectively collected data from patients with QAV undergoing TAVR in our center and conducted a systematic literature review for further investigation.

**Results:** Five patients with QAV were treated with TAVR between April 2016 and December 2023. The median age was 67 years (range: 59–86), and the median Society of Thoracic Surgeons score (STS-score) was 3.750% (range: 0.916%–11.823%). Procedural success was achieved in all cases. The median follow-up period was 3 years (from 30 days to 7 years). Four of the patients exhibited no serious complications, while one experienced delayed coronary obstruction. Our systematic review included 31 cases from 21 publications and our center. The median age of patients was 79 years (range: 57–90), including 18 males. The median STS score was 7.835%. Severe AS was present in 64.5% of the patients and severe AR in 41.9%. The most common QAV subtype was type B (48.4%). Technical success was achieved in 100% of the cases, with two cases reporting coronary obstruction and one required a permanent pacemaker implantation. During a median follow-up period of 1 year (from 30 days to 7 years), one case experienced serious complications of delayed coronary obstruction.

**Conclusion:** The TAVR may be an alternative treatment for patients with QAV, preliminarily demonstrating feasible early and long-term results from current experience. However, extra precautions regarding coronary artery obstruction complications are necessary due to the rarity and anatomical complexity of QAV.

## 1. Introduction

Quadricuspid aortic valve (QAV) is a congenital aortic valve malformation with a prevalence rate of 0.003%–0.05% [1, 2]. Patients with QAV face increasing risks of aortic regurgitation (AR) and aortic stenosis (AS) as they grow, with progressive AR being more common. Surgical aortic valve replacements (SAVR) have traditionally been the primary treatment for patients with severe AS or AR. Recently, transcatheter aortic valve replacement (TAVR) has been similarly recommended for patients with severe calcific AS according to the 2020 AHA/ACC guideline [3]. The TAVR has become the preferred treatment for tricuspid aortic valve, and evidence supporting its safety and feasibility in patients with congenital bicuspid aortic valve abnormalities is accumulating [4–8]. However, randomized, controlled trials have excluded QAV, and SAVR may increase complications and mortality in high-risk patients [9–11]. Using a less invasive transcatheter therapy in this high-risk population became rapidly attractive. Despite the low prevalence of QAV, a few cases of QAV treated with TAVR have been reported, indicating promising outcomes [12]. The limited evidence and the demand for less invasive treatments have highlighted the need for further review and study. Consequently, we performed this case series and systematic review to evaluate TAVR in patients with QAV.

## 2. Materials and Methods

### 2.1. Patient Population

For these case series, we reviewed our institutional heart valve database from April 2016 to December 2023, which included 1600 cases, to identify patients with QAV who underwent TAVR. Before the procedure, all patients with QAV were identified using transthoracic echocardiography (TTE) and electrocardiography-gated contrast-enhanced multidetector computed tomography (MDCT) examination. The QAVs were categorized according to Hurwitz and Roberts's classification [13]. The dimensions of the aortic annulus, aortic root, ascending aorta, and left ventricular outflow tract were also measured. After a comprehensive evaluation of the patient's ages, comorbidities, frailty, anatomical features, and propensity, our heart team recommended that all five retrieved patients undergo the TAVR procedure. Data, including the baseline characteristics, procedural variables, and follow-up data, were stored in the database.

**Figure 1:**
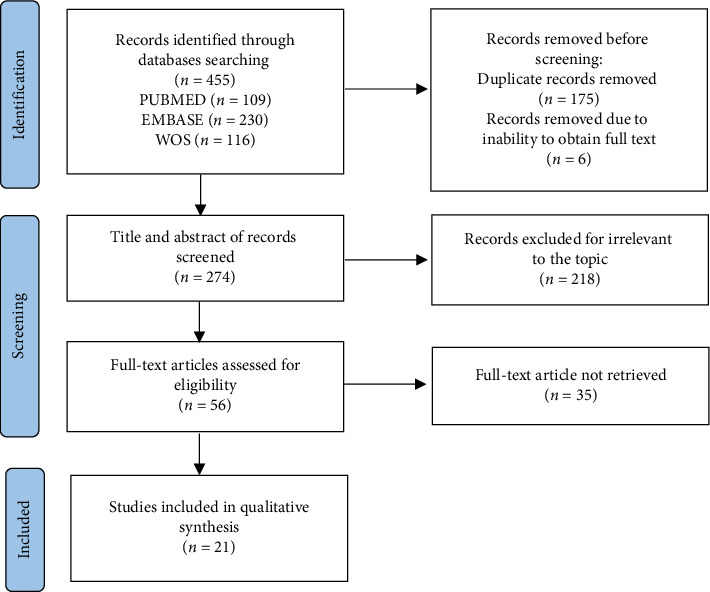
PRISMA flow diagram. Inclusion criteria: cases with QAV and treated with TAVR acquired according to the keywords. The exclusion criteria were as follows: pediatric and veterinary cases, abstracts and articles written in languages other than English, review articles or articles without actual case reports, and cases with non-QAV or underwent SAVR.

In addition, we conducted a systematic review by searching for published articles from January 2002 to May 2024. PubMed, EMBASE, and Web of Science were searched by two independent authors (W.S. and D.Z.) using the following keywords: (TAVR or TAVI or (transcatheter aortic) or (transfemoral aortic) or (transsubclavian aortic) or (transapical aortic) or (percutaneous aortic) or (heart valve prosthesis implantation) and (quadricuspid). The systematic search revealed 455 publications for possible inclusion. After removing duplicates, the remaining titles and abstracts were reviewed, and irrelevant publications were excluded. Of the remaining 56 publications, full-text articles were obtained for more details, and 35 were excluded. The systematic review was conducted following the *Preferred Reporting Items for Systematic Reviews and Meta-Analyses* (PRISMA) guideline [14], and the search strategy is depicted in Figure 1. Ultimately, 21 articles [12, 15–30] were eventually included to collect detailed data. Due to the small sample size of the identified cases, data were tabulated and summarized without further formal statistical analysis.

### 2.2. Procedural Details

All procedures were determined by our multidisciplinary heart team. General or local anesthesia was used during the TAVR procedure. The J-Valve prosthesis (Jiecheng, Suzhou, China) was implanted through a transapical approach [31, 32]. The VenusA‐Valve (Venus Medtech, Hangzhou, China), VenusA-Plus (Venus Medtech, Hangzhou, China), and Sapien XT (Edwards Lifesciences, Irvine, California) were implanted through a preferred transfemoral access. Balloon predilatation was routinely performed for patients with severe AS, while postdilatation was performed at the operators' discretion. Other procedural details have been described previously [26, 33, 34].

### 2.3. Statistical Analyses

Continuous variables are presented as the median (minimum–maximum). Categorical data are presented as numbers (%). Statistical analyses were conducted using SPSS 26.0 software (IBM SPSS Statistics for Macintosh, Version 26.0. IBM Corp, Armonk, NY, USA).

## 3. Results

### 3.1. Case Series of Our Center

The case series enrolled three consecutive patients and two from our center with updated follow-up data. These two patients were previously reported in 2021 [26]. Detailed baseline characteristics and comorbidities are displayed in Table 1. The median age of patients was 67 years (59–86), with four males. The median Society of Thoracic Surgeons score (STS-score) was 3.750% (1.180%–11.823%), indicating all patients were at intermediate or high risk of surgery, with four patients classified as New York Heart Association (NYHA) Functional Class III or IV. All patients experienced cardiovascular system comorbidities: four patients with hypertension and two with coronary artery disease. The echocardiography revealed severe AS combined with moderate or severe AR in two patients, severe AR with or without AS in two patients, and moderate AS and AR in one patient who underwent TAVR due to age and frailty. The median left ventricular ejection fraction (LVEF) evaluated by echocardiography was 64.3% (43.8%–72.9%). One patient experienced significant left ventricular enlargement with an LVEF of 43.8%, while the other patients with LVEFs were within the normal range.

**Figure 2:**
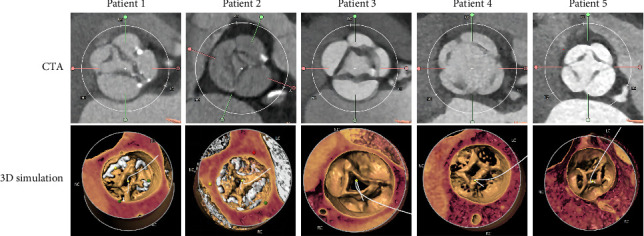
The anatomical structures of the QAVs of five patients were demonstrated using computed tomography angiography (CTA) and three-dimensional (3D) simulation.

**Figure 3:**
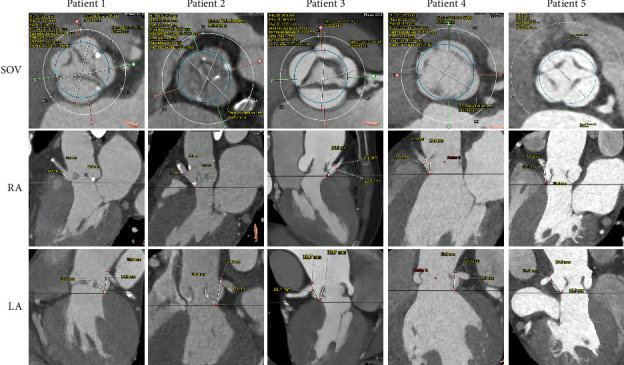
The dimensions of the aortic sinus, coronary artery, and valve leaflet of the QAVs of five patients were measured by CTA. (SOV) Measurements of the anticipated morphology of the implanted valve after release at the aortic sinus level and the diameters of right coronary sinus (RC) and left coronary sinus (LC); (RA) Assessment of right coronary artery (RCA) occlusion risk, including the heights of the RCA and the lengths of the adjacent valve leaflet; (LA) Assessment of left coronary artery (LCA) occlusion risk, including the heights of the LCA and the lengths of the adjacent valve leaflet.

All cases were identified as QAV by MDCT: two were type C, two were type E, and the remaining one was type B (Figure 2). The data of preoperative MDCT measurements are presented in Table 2. The median diameter of the aortic annular was 24.4 mm (21.3–29.5). The data of the aortic valvular complex were measured following the *Expert Consensus Document of the Society of Cardiovascular Computed Tomography* (Figure 3) [35]. The diameter of the sinus and the height of the coronary artery were measured to assist in assessing the risk of coronary ostium occlusion. Two patients were identified as at high risk for coronary ostia obstruction. The patient with severe AR was not considered for intraoperative coronary protection due to a broad coronary sinus and lack of calcification.

**Figure 4:**
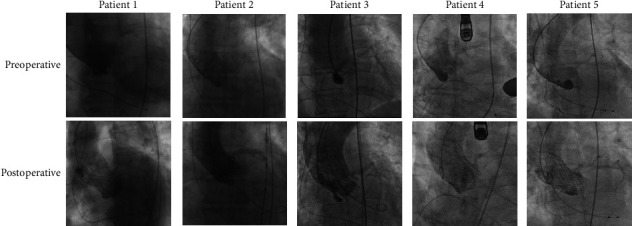
Preoperative and postoperative DSA imaging of transcatheter aortic valve replacement (TAVR) in the five patients included with a QAV.

The procedural data and the follow-up performance are indicated in Table 3. The device implantation was achieved in all cases (Figure 4). One patient was implanted with a 29 mm J-Valve prosthesis. Two patients received a VenusA prosthesis with dimensions of 29 and 26 mm, another patient received a 29 mm VenusA-Plus prosthesis, and the remaining patient was implanted with a 26 mm Sapien XT. Local anesthesia was used for the four patients who underwent transfemoral TAVR, while one patient underwent transapical TAVR under general anesthesia. Predilatation was performed in two patients with severe AS. No abnormalities were found after balloon pre-extension for the one with lower coronary height. Consequently, coronary protection was not implemented after careful consideration. The patient with moderate AR and AS underwent postdilatation due to moderate paravalvular leak (PVL) during the procedure, resulting in minor residual PVL postoperatively. The other four patients experienced no PVL being detected. One patient developed a prolonged QRS interval and was reported with an in-hospital complication of a new left bundle branch block without the need for permanent pacemaker implantation. With a median follow-up period of 3 years (from 30 days to 7 years), four patients reported significant improvement in heart failure symptoms without any major complications. Two patients [26] completed their long-term follow-ups of 6 and 7 years, achieving the longest follow-up periods reported. However, the patient who underwent transapical TAVR experienced delayed coronary ostium occlusion on the 25^th^ postoperative day. He experienced refractory heart failure and required intensive care in the intensive care unit. Subsequently, he received surgery to remove the initially implanted valve prosthesis and was awaiting a heart transplant.

### 3.2. TAVR in Patients With QAV: A Systematic Review of the Literature

Between January 2010 and March 2023, 28 cases from 21 publications reported TAVR treatment for symptomatic QAV diseases (Table 4). Besides, we included three new cases from our center and provided updates on two patients previously reported by Zhou et al. [26] Data on identified patient characteristics and clinical outcomes are tabulated and summarized in Table 5. The median patient age was 79 (57–90), with 58.1% male. A total of 19 patients were reported NYHA grading, and all were ≥ grade III. The STS scores were reported for 14 patients, with a median score of 7.835% (1.180%–18.14%). Twenty-two patients were reported with comorbidities, including coronary artery disease (50%), hypertension (45.5%), and heart failure (22.7%) at the highest rates. All patients underwent echocardiography to assess the functional status of the aortic valve. Twenty patients (64.5%) were confirmed with severe AS, while 13 (41.9%) experienced severe AR. Preprocedural cardiac CTA scans were conducted in all cases reported, and congenital QAV was revealed. The most common QAV subtype was type B (48.4%), followed by type A (22.6%) and type C (16.1%), according to the Hurwitz and Roberts classification for QAV (Supporting Tables 1 and 2).

Procedural success was achieved in all patients. Thirty cases were reported with the TAVR device and the valve size chosen. The frequency of self-expandable valve (15/31) implantation was comparable to balloon-expandable valves (16/31) in the case series. The median size of the self-expandable valve in 16 cases was 27 mm (23–32), and the balloon-expandable valve in 14 cases was 23 mm (23–26). Six patients with pure AR and one patient with severe AR combined with minor AS underwent implantation of a J-Valve through a transapical approach. The remaining 23 patients experienced severe AS with various degrees of AR. Of these, 11 were implanted with the Edwards Sapien 3 valve, two with the Edwards Sapien XT valve, four with the VenusA valve, and the other six with the Edwards Sapien valve, VenusA-Plus, Evolut-R, Evolut FX, Medtronic CoreValve, and PROTICO. All of these were implanted through the transfemoral approach, except for one case reported by Blanke et al. in 2011, which involved the implantation of the Edwards Sapien valve through a transapical approach [15].

No severe procedural complications occurred in 27 cases. Four cases demonstrated complications that were promptly remedied. In Liu et al.'s case report, one patient received a permanent pacemaker due to a high-degree atrioventricular block 3 days later and another patient with pure AR received a blood transfusion due to a hemorrhage at the apex during the procedure [12]. In the case presented by Takahashi et al., a patient developed an occlusion of the left main coronary artery immediately after device expansion despite the routine preoperative assessment, which did not suggest a significant risk of coronary ostium occlusion with a 23 mm balloon-expandable transcatheter heart valve (THV) [22]. Subsequently, a stent was deployed, resulting in reperfusion and stable hemodynamics. Another exceptional patient in Han's case report needed a second valve implanted due to severe PVL after the initial implantation [25].

Moreover, two cases recently performed coronary protection by parking a stent and achieved considerable outcomes [36, 37]. Follow-up results were reported in 19 cases, the longest being 7 years. One case from our center experienced major complications with refractory heart failure symptoms due to delayed coronary artery occlusion. In Liu's case series, the median follow-up period was 18 months (12–56), without deaths occurring during the 1-year follow-up and significant improvement in heart failure symptoms [12]. The two cases reported in Zhou et al.'s article [26] updated their follow-up data in our case series, demonstrating the outcomes of 6- and 7-year follow-ups, respectively, without valve structure deterioration or major adverse cardiovascular events occurring during the period. The procedural details are summarized in Supporting Table 3.

## 4. Discussion

The current case series, including five patients with QAV who underwent TAVR, is the first cohort reporting prolonged follow-up data, with three cases presenting follow-up outcomes of 3 years or more. To better understand the effects of treating patients with QAV with TAVR, we performed a systematic review, including 28 cases from 21 published studies and 5 cases (three new cases reported for the first time and two published cases with updated follow-up data) from our center. This is the most updated and comprehensive systematic review of the use of TAVR in patients with QAV. All QAV cases treated with TAVR in this study were procedurally successful, without deaths occurring during the procedure. Follow-up data were specified for 19 patients (19/31, 61.3%), and eight cases with more than 12 months of follow-up indicated promising results. Despite considerable heterogeneity across studies or the small sample size, our results suggest that TAVR is technically feasible in selected QAV patients with AS, AR, or both. Early or long-term morbidity and mortality are acceptable, except for one case of delayed coronary obstruction resulting in refractory heart failure, attributable primarily to the unique anatomy of the QAV. However, specific anatomical and pathophysiological characteristics, limited procedural experience, the lack of dedicated valves, and other factors pose challenges that require more in-depth practice and research to be resolved.

The QAV is a rare congenital cardiac defect, with an estimated frequency of less than 0.05% in the general population [1, 2]. Previous reports have indicated that QAV is frequently associated with progressive AR, while AS and ascending aortic enlargement are less common [1]. Nearly one-half of the patients with QAV require surgical procedures for their aortic valve disease. Historically, surgical replacement or repair has been the standard treatment for QAV with AS or AR. However, a significant proportion of patients with advanced age, multiple comorbidities, and frailty are considered high risk for surgery and are often excluded from surgical treatment. The TAVR has emerged as a treatment alternative for TAV patients with symptomatic severe AS, irrespective of the surgical risk profile, owing to its favorable safety and efficacy profile [9–11]. The broad implementation of TAVR in patients with a wide range of anatomic diversity has led to the exploration of its feasibility for TAVR in off-label indications such as severe AR, anomalous valvular anatomy, and degenerated surgical bioprostheses, including individuals with QAV [3, 40]. Treating aortic valve diseases in patients with QAV using TAVR presents unique challenges regarding technical feasibility. In decision-making for TAVR, it is crucial to carefully assess the QAV anatomy and possible coexisting congenital abnormalities such as intracardiac shunts, coronary anomalies, aortic dilatation, and other valve disorders [41]. Accordingly, detailed multimodality imaging for preoperative assessment is required, including TTE, trans-esophageal echocardiography, cardiovascular CT, and cardiovascular magnetic resonance [18, 42, 43].

The QAV anatomy should be precisely gauged. As demonstrated in several studies, TTE is a noninvasive technique, but QAV may not always be well visualized with TTE. As a result, it may be better suited as a screening modality for QAV. TEE is utilized if further investigation is warranted or TTE results are suboptimal [44]. The TEE is considered a novel transillumination mode for diagnosing QAV and evaluating excoriated aortic pathologies.

Besides, TEE is an effective means of intraoperative detection, allowing better visualization of the valve location, functional status of the implanted bioprosthetic valves, and assessment of PVL [45]. The case series in our center suggests that TEE is superior to TTE for clarifying aortic valve morphology, with three patients correctly identified by TEE compared to only one by TTE. Preoperative preparations typically include cardiac CTA scans, routinely performed before TAVR for indication screening and strategic planning. All cases enrolled in our study and reported completed cardiac CTA [12]. The CT evaluations provide detailed anatomical features of the aortic root and leaflets and offer quantitative measurements and preprocedural simulations. These simulations guide preoperative valve selection and predict intraoperative complications such as annular rupture, coronary artery occlusion, and the probability of permanent pacemaker implantation [20, 23, 25]. All cases were identified as QAVs by CTA, and further classification of QAV also depends on CTA imaging.

Based on the relative size of the supernumerary cusp, the Hurwitz and Roberts classification divides QAVs into seven types from A to G, and it is the most popular standard models [13]. Previous studies have demonstrated that the three most common QAV subtypes are types A, B, and C [1, 2, 46], consistent with the data from our systematic review. The abnormal ridge distribution is associated with high procedural complexity and an increased risk of perioperative complications [23]. Fukui et al.'s case report described a successful experience with unequal QAV stenosis treated with TAVR, highlighting that interventional cardiologists should particularly recognize the detailed anatomic relationships among the four cusps and aberrant coronary artery origin. This is crucial to avoid inappropriate transcatheter valve deployment or coronary complications during TAVR for QAV [23, 47].

Coronary artery occlusion is a major complication of TAVR. The risk factors for coronary artery occlusion in TAV patients undergoing TAVR include smaller valsalva, low coronary height, and bulky valve leaflet calcification on CT [48–50]. However, if the risk factors of coronary artery occlusion for QAV with TAVR were deemed to be similar to those for the TAV, the patients reported by Takahashi et al. and the similar case in our center were not considered at high risk for coronary ostium occlusion based on preoperative multimodality imaging evaluation. Despite this, life-threatening complications occurred coincidentally. Therefore, the risk of coronary artery occlusion is idiosyncratic and theoretically higher in QAV than in TAV stenosis. The QAV significantly differs from TAV in the morphologies of the aortic sinus, the origin of the coronary arteries, the length of each valve leaflet, and the distributions of calcifications. In Takahashi et al.'s study, TAVR carries a higher risk of coronary artery occlusion in patients QAV than in TAV due to the greater leaflet height and shallower cusp depth [22]. In addition, in Fukui's report, the complex anatomical structure of four cusps and aberrant coronary artery origin were promptly identified, and coronary artery protection was taken timely to avoid this serious complication [23].

Moreover, caution is warranted as coronary occlusive events can occur not only during the procedure but also in a delayed state, potentially causing severe consequences if not recognized in time. The additional case in our series introduced a delayed coronary artery occlusion of the left anterior descending artery, which was not recognized until coronary angiography was performed 1 month after the procedure. We suggest that this was a critical factor in the patient's significantly adverse prognosis. Accordingly, more attention should be paid to the preoperative assessment for TAVR in patients with QAV, avoiding the solidified pre-TAVR assessment methods used for patients with TAV for others with QAV. Key aspects of preoperative evaluation should include measurements of sinus morphology, the location of the coronary ostium, and the length of every separate leaflet. Besides, the perioperative period should be carefully managed, with timely and detailed coronary angiography after the prosthetic valve deployment being essential [51]. Close attention should be paid to specific clinical symptoms, such as typical ST-segment elevation ventricular fibrillation and heart failure symptoms, to help in the early identification of delayed coronary events.

Although QAV has been detected more frequently and TAVR has expanded its indications to include QAV-associated valvular disease, there are no specific TAVR guidelines or devices designed and manufactured for patients with QAV. Consequently, new conduction disturbances or arrhythmias requiring permanent pacemaker or implantable cardioverter-defibrillator implantation and severe PVL—these frequent complications of TAVR in TAV patients—should not be disregarded in patients with QAV [52]. In Han et al.'s case report, a self-expanding valve of matching size was selected according to the conventional TAV evaluation principle and located following the routine of the TAV-TAVR procedure. However, severe PVL occurred after the first attempt, necessitating a second valve implantation of 10 mm higher. This case highlights the importance of patient-specific procedural decisions for individuals with rare or complex aortic root pathology, particularly in valve size selection and implantation depth [25].

Meanwhile, patients with QAVs may be correlated with an elevated risk of AR [31]. Jena-Valve and J-Valve are new-generation self-expandable valves that are considered a feasible option for transcatheter treatment of noncalcified native valve and pure AR, as they are anchored using a clipping mechanism against the native aortic valve leaflets [12, 53–55]. Luo et al. reported a similar case in which pure AR was treated successfully with this device before [24]. This occurrence may have been coincidental and requires additional investigation in the future. However, the challenges of implanting these devices persist. During the procedure, it is difficult to position the clasper in the individual sinus of QAV's four sinuses. Consequently, preoperative assessment and strategic planning for device location in QAV are essential to avoid clasper blockage by the leaflet commissure [12, 24].

Despite advancements in devices and preoperative evaluation, challenges in clinical diagnosis, device selection, coronary risk assessment, and implantation strategies persist in patients with QAV. Traditional preoperative assessment for TAVR remains inadequate. The emerging image-based techniques, such as 3D printing and computational fluid dynamics [56], offer new solutions for the management of QAV. The 3D printing enables detailed reconstruction of aortic root models with patient-specific anatomical variations, allowing for precise visualization of the aortic valve leaflets, calcification, and sinuses [57]. Liu et al. demonstrated that 3D printing was used for preoperative simulation, enhancing the understanding of QAV malformations and aiding in predicting coronary complications in high-risk patients [12]. The 3D-printed models and in vitro simulation have proven valuable in guiding procedural planning, particularly in complex cases, which could help accurately select THVs and predict relevant procedural complications [56, 58]. In the future, this technology has the potential to address the challenges of TAVR in QAV patients and help develop more tailored surgical strategies. The pulsatile 3D-printed simulation platform and the computational fluid dynamics can accurately replicate the human hemodynamic environment [59, 60]. Integrating these methods provides a realistic in vitro platform for testing and developing devices, enabling precise simulation of cardiac tissue stress, deformation, and blood flow dynamics [59, 61]. With ongoing technological advancements and decreasing costs, the adoption of 3D printing and computational fluid dynamics is expected to enhance the development of TAVR technology and the management of QAV.

Lastly, a well-established postoperative follow-up strategy is crucial for TAVR-treated patients with QAV due to the lack of strong evidence for these off-label indications. At our center, we have successfully implemented the hospital–community–home closed-loop management model. In addition to standardized follow-up staff to ensure patients' timely postoperative assessment, we recommend home-appropriate smart wearable devices for selected patients. This smart wearable device helps monitor patients' cardiac activity, sleep, respiration, oxygen saturation, heart rate variability, and exercise, uploading data to our data management center for timely identification of adverse events [62, 63]. Our hospital and the community provide comprehensive recovery tracking and specialized technical guidance to safeguard the patient's prognosis. Consequently, TAVR procedures for patients with QAV can be smoothly and effectively implemented at our institution based on this robust management model.

## 5. Limitations

Our cases and the previously reported cases of treating symptomatic QAV diseases with TAVR were feasible. However, there are significant limitations to consider when interpreting these successes. First, this study was conducted on a small cohort at our single center, and the relatively small number of patients hinders us from making further convincing conclusions. The total sample size of the systematic review is 31, which is small for effective inferences. Second, the available data are based solely on case reports and do not include prospectively designed trials with prospectively included patients and defined outcome parameters. Third, the duration of follow-up among the reported cases varies considerably, and no systematic data on long-term outcomes are available. Finally, the possibility of positive reporting or publication bias cannot be excluded, resulting in cases with poor outcomes not reported in the literature.

## 6. Conclusion

Our institution with TAVR in QAV demonstrates preliminary feasible and acceptable early and long-term outcomes. The complex anatomical and pathophysiological features demand that TAVR in patients with QAV requires a more in-depth preprocedure review and patient-specific procedural decisions, especially regarding valve size selection and depth of implantation. Furthermore, prompt coronary risk identification and aggressive adoption of coronary protection strategies may be advocated. Larger sample size studies and further follow-up are essential to demonstrate the effectiveness and sustainability of this intervention.

## Figures and Tables

**Table 1:** Preoperative clinical characteristics.

Patients	Sex	Age (year)	BMI (kg/m^2^)	NAHY FC	STS-score (%)	6MWT-length (m)	Comorbidities	Functional status of aortic valve	Aortic valve morphology by TTE/TEE	LVEF (%)	Otherwise specified
Patient 1⁣^∗^	Male	79	21.7	IV	6.330	324	Hypertension, coronary artery disease	Moderate AS, moderate AR	TAV/TAV	57.1	NA
Patient 2⁣^∗^	Male	86	27.4	IV	11.823	289	Hypertension, cerebral infarction	Severe AS, moderate AR	TAV/TAV	64.3	NA
Patient 3	Female	66	19.5	II	3.750	324.2	Coronary artery disease, chronic obstructive pulmonary disease	Severe AS, severe AR	QAV/QAV	72.9	NA
Patient 4	Male	59	32.6	III	9.160	480	Hypertension, diabetes mellitus	Severe AR	NA/QAV	43.8	LVIDd 8.00 cm
Patient 5	Male	67	22.6	II	1.180	385	Hypertension, dyslipidemia	Severe AR, mild AS	TAV/QAV	64.5	NA

Abbreviations: 6MWT, 6-min walk test; AR, aortic regurgitation; AS, aortic stenosis; BMI, body mass index; LVEF, left ventricular ejection fraction; LVIDd, left ventricular end-diastolic diameter; NA, not available; NYHA FC, New York Heart Association functional class; QAV, quadricuspid aortic valve; STS, Society of Thoracic Surgeons; TAV, tricuspid aortic valve; TEE, transesophageal echocardiography; TTE, transthoracic echocardiography.

⁣^∗^Cases reported by Zhou et al. [26] with updated data (Zhou D et al. *J Invasive Cardiol*. 2021; 33:E839–E840).

**Table 2:** Preoperative computed tomography (CT) measurements.

Patients	QVA type (Hurwitz and Roberts)	Calcification	Annulus diameter (mm)	Annulus area (mm^2^)	Sinus diameter (mm)	STJ diameter (mm)	Ascent aorta diameter (mm)	RCA height (mm)	LCA height (mm)
Patient 1⁣^∗^	E	Moderate	25.6	496.8	RC:40.0 LC:35.5	30.55	37.8	10.3	14.6

Patient 2⁣^∗^	B	Moderate	24.0	441.3	RC:31.9 LC:34.7	31.5	32.9	17.7	14.5

Patient 3	C	Mild	24.4	462.3	RC:39.6 LC:35.8	34.3	44.4	6.9	13.6

Patient 4	C	None	29.5	670.6	RC:37.1 LC:39.0	31.5	39.4	9.5	9.8

Patient 5	E	Mild	21.3	357.4	RC:32.1 LC:32.9	28.1	35.6	14.0	11.6

Abbreviations: AD, average diameter; LC, left coronary; LCA, left coronary artery; QAV, quadricuspid aortic valve; RC, right coronary; RCA, right coronary artery; STJ, sinotubular junction.

⁣^∗^Cases reported by Zhou et al. [26] with updated data (Zhou D et al. *J Invasive Cardiol*. 2021; 33:E839–E840).

**Table 3:** Procedural clinical characteristics and follow-up data.

Patients	Approach	TVAR device used	Valve size	Anesthetic type	Procedural outcomes	Predilatation	Postdilatation	Procedural complications	30-day complication	Follow-up duration	1-year NYHA class	1-year complication
Patient 1⁣^∗^	TF	VenusA	29	Local	Success	No	Yes	Mild PVL	None	7 years	III	Mild PVL
Patient 2⁣^∗^	TF	Sapien XT	26	Local	Success	Yes	No	None	Minor bleeding	6 years	II	None
Patient 3	TF	VenusA	26	Local	Success	Yes	No	Prolong QRS interval	New LBBB	3 years	I	None
Patient 4	TAp	J-valve	29	Generate	Success	No	No	None	Delayed coronary obstruction	0.5 years	IV	Bioprosthetic valve removed; recurrent heart failure and required heart transplantation
Patient 5	TF	VenusA-plus	26	Generate	Success	No	No	None	None	30 days	NA	NA

Abbreviations: LBBB, left bundle branch block; LVEF, left ventricular ejection fraction; NA, not available; PVL, paravalvular leak; TAp, transapical; TAVR, transcatheter aortic valve replacement; TF, transfemoral.

⁣^∗^Cases reported by Zhou et al. with updated data (Zhou D et al. *J Invasive Cardiol*. 2021; 33:E839–E840).

**Table 4:** Summary of the literature on QAV.

First author and year of publication	Sex	Age (year)	NYHA FC	Functional status of aortic valve	QVA type (Hurwitz and Roberts)	TVAR device used (valve size, mm)	Approach	Procedural outcomes	Procedural/In-hospital complications	Follow-up duration
Blanke et al. 2011 [15]	Female	79	NA	Severe AS, moderate AR	E	Edwards Sapien (26 mm)	TAp	Success	None/NA	NA

Yu and Lee. 2014 [16]	Male	80	IV	Severe AS, moderate AR	A	Edwards Sapien XT (26 mm)	TF	Success	None/NA	NA

Bruschi, and De Marco, and Klugmann 2014 [30]	Male	78	NA	Severe AS, moderate AR	A	Medtronic CoreValve (29 mm)	TF	Success	Trivial PVL/NA	NA

Sidharta et al. 2015 [17]	Male	90	III	Severe AS, moderate AR	B	PORTICO THV (a self-expanding, and repositionable valve) (27 mm)	TF	Success	Minor PVL/None	1 month

Ibrahim et al. 2018 [18]	Female	82	NA	Severe AS, moderate AR	A	Edwards Sapien 3 (23 mm)	TF	Success	None/None	NA

Tohoku 2018 [19]	Female	85	NA	Severe AS, moderate AR	C	Edwards SAPIEN 3 (23 mm)	TF	Success	Trivial PVL/None	NA

Aoyama 2019 [20]	Male	83	NA	Severe AS, severe AR	B	Evolut R (29 mm)	TF	Success	None/None	NA

Benkemoun, Bramlage, and Beaufigeau 2020 [21]	Female	87	NA	Severe AS	A	Edwards SAPIEN 3 (23 mm)	TF	Success	None/None	6 months

Takahashi et al. 2020 [22]	Female	84	NA	Severe AS, severe AR	A	Edwards SAPIEN 3 (23 mm)	TF	Success	Hemodynamically unstable just after the prosthetic valve was deployed. Found 99% stenosis of the left main coronary artery acquired reperfusion and stable hemodynamics after a stent deployed./NA	NA

Fukui et al. 2020 [23]	Female	74	NA	Severe AS, moderate AR	B	Edwards SAPIEN 3 (23 mm)	TF	Success	None/NA	3 months

Luo et al. 2021 [24]	Male	62	III-IV	Minor AS, severe AR	A	J-valve (27 mm)	TAp	Success	None/None	6 months

Han et al. 2021 [25]	Male	70	NA	As, AR	B	VenusA (26 mm)	TF	The first valve with severe PVL, and the second one (implanted 10 mm higher) with moderate PVL	Moderate PVL/NA	NA

Zhou et al. 2021 [26]	Male	79	NA	Severe AS, moderate AR	E	VenusA (23 mm)	TF	Success	None/None	5 years
	Male	86	NA	Severe AS, moderate-to-severe AR	B	Edwards SAPIEN XT (26 mm)	TF	Success	None/None	3 years

Mukherjee et al. 2021 [36]	Female	75	III	Severe AS	B	Edwards SAPIEN 3 (23 mm)	TF	Success	RCA occlusion by displaced leaflets after predilation and a coronary stent parked for protection/None	1 month

Liu et al. 2022 [12]	Male	82	IV	Severe AR	A	J-valve (27 mm)	TAp	Success	Bleeding and transfusion needed/None	Median follow-up period: 18 (12–56) months
	Male	72	IV	Severe AR	F	J-valve (29 mm)	TAp	Success	None/None	NA
	Female	71	III	Severe AR	B	J-valve (27 mm)	TAp	Success	None/none	None
	Male	69	III	Severe AR	D	J-valve (29 mm)	TAp	Success	Trivial PVL/permanent pacemaker implantation for advanced A-V block	6 months
	Male	75	III	Severe AS, moderate AR	B	VenusA (32 mm)	TF	Success	None/None	4 years

Ochiai et al. 2022 [27]	Female	75	NA	Severe AS, mild AR	C	Edwards SAPIEN 3 (23 mm)	TF	Success	None/None	NA

Melania et al. 2022 [37]	85	III	Severe AS, moderate AR	B	A balloon-expandable valve (NA)	NA		Success	Coronary protection and a chimney stenting performed after implantation/None	6 months

Zhang et al. 2023 [28]	Male	57	NA	Severe AR	B	J-valve (29 mm)	TAp	Success	Minor PVL/None	6 months

Sato et al. 2023 [29]	Female	83	NA	Severe AS, moderate AR	B	Edwards Sapien 3 (23 mm)	TF	Success	None/None	None
	Female	75	NA	Severe AS, mild AR	C	Edwards Sapien 3 (23 mm)	TF	Success	None/None	Follow-up duration

Aquino-Bruno et al. 2024 [38]	Male	81	3	Severe AS, moderate AR	B	Edwards Sapien 3 (23 mm)	TF	Success	None/None	NA
	Male	79	3	Severe AS, mild AR	B	Edwards Sapien 3 (23 mm)	TF	Success	None/None	NA

Bienstock et al. 2024 [39]	Male	80	2.5	Severe AS, moderate-to-severe AR	B	Evolut FX (26 mm)	TF	Success	None/NA	NA

Abbreviations: AR, aortic regurgitation; AS, aortic stenosis; NA, not available; NYHA FC, New York Heart Association functional class; PCI, percutaneous coronary intervention; PVL, paravalvular leak; QAV, quadricuspid aortic valve; TAp, transapical; TAVR, transcatheter aortic valve replacement; TF, transfemoral.

**Table 5:** Characteristics and clinical outcomes of all cases involved with QAV underwent TAVR in the systematic review.

	Values for data available	Data available (N)
Baseline patient characteristics		
Age	79 (57–90)	31
Male	18 (58.1%)	31
NYHA FC	III (III-IV)	19
STS-score	7.835% (1.180%–18.14%)	14
Comorbidities		
Hypertension	10 (45.5%)	22
Coronary artery disease	11 (50.0%)	22
Heart failure	5 (22.7%)	22
Diabetes mellitus	2 (9.1%)	22
Cerebrovascular disease	3 (13.6%)	22
Dyslipidemia	1 (4.5%)	22
Chronic obstructive pulmonary disease	3 (13.6%)	22
Atrioventricular blockage	1 (4.5%)	22
Arrhythmia	2 (9.1%)	22
Echocardiography data		
Severe AS	20 (64.5%)	31
Severe AR	13 (41.9%)	31
LVEF	48.5% (33%–72.9%)	16
QVA type		
A	7 (22.6%)	31
B	14 (45.2%)	31
C	5 (16.1%)	31
D	1 (3.2%)	31
E	3 (9.6%)	31
F	1 (3.2%)	31
Periprocedural data		
TAVR device used		
Self-expandable valve	16 (51.6%)	31
Balloon-expandable valve	15 (48.4%)	31
Valve size (mm)		
Self-expandable valve	27 (23–32)	16
Balloon-expandable valve	23 (23–26)	14
Approach		
TF	22 (73.3%)	30
TAp	8 (26.7%)	30
Procedural outcomes		
Procedural success	31 (100.0%)	31
Procedural complications		
Paravalvular leak (≥ moderate)	1 (3.2%)	31
Coronary obstruction	1 (3.2%)	31
Bleeding	1 (3.2%)	31
New conduction disturbances and arrhythmias	1 (3.2%)	31
Follow-up outcomes		
Follow-up duration	1 year (1 month–7 years)	19
Complications	1 (5.3%)	19

*Note:* Values are median (min–max), *n* (%), or *n*.

Abbreviations: AR, aortic regurgitation; AS, aortic stenosis; LVEF, left ventricular ejection fraction; NYHA FC, New York Heart Association functional class; QAV, quadricuspid aortic valve; STS-score, Society of Thoracic Surgeons Score; TAp, transapical; TAVR, transcatheter aortic valve replacement; TF, transfemoral.

## Data Availability

The data that support the findings of this study are available from the corresponding author upon reasonable request.

## Nomenclature

QAV: Quadricuspid aortic valve
AR: Aortic regurgitation
AS: Aortic stenosis
TAVR: Transcatheter aortic valve replacement
TAVI: Transcatheter aortic valve implantation
STS-score: Society of Thoracic Surgeons Score
PVL: Paravalvular leak
SAVR: Surgical aortic valve replacement
TAV: Tricuspid aortic valve
THV: Transcatheter heart valve
TTE: Transthoracic echocardiography
MDCT: Electrocardiography-gated contrast-enhanced multidetector computed tomography
NYHA: New York Heart Association
LVEF: Left ventricular ejection fraction
TEE: trans-esophageal echocardiography
PPM: Prosthesis-patient mismatch
ACC: American College of Cardiology
AHA: American Heart Association
6-MWT: A six-minute walk test
LC: Left coronary
LCA: Left coronary artery
RC: Right coronary
RCA: Right coronary artery
CTA: Computed tomography angiography

## Supporting Information

Supporting Table 1: List of all case reports of transcatheter aortic valve implantation in patients with QAV. Patient characteristics. Supporting Table 2: List of all case reports of transcatheter aortic valve implantation in patients with QAV. Preoperative CT measurements. Supporting Table 3: List of all case reports of transcatheter aortic valve implantation in patients with QAV. Periprocedural and Follow-up data.
