# Supplementary material for: Transcatheter Aortic Valve Replacement in Patients With Quadricuspid Aortic Valve: A Case Series and Systematic Review
Source: Cardiol Res Pract. 2025 Feb 5;2025:7815279. doi: 10.1155/crp/7815279 (PMC11824809; doi:10.1155/crp/7815279)
Supplement: Supporting Information — Additional supporting information can be found online in the Supporting Information section. [file 7815279.f1.docx]

**Appendix files**

**Supplementary Table 1. List of all case reports of transcatheter aortic valve implantation in patients with QAV. Patient characteristics.**

| First author and  year of publication | Sex | Age (year) | NYHA FC | STS-score/EuroSCORE | Comorbidities | Functional  status of aortic  valve | | QVA type  (Hurwitz and  Roberts) | | Otherwise Specified |
| --- | --- | --- | --- | --- | --- | --- | --- | --- | --- | --- |
| Blanke et al. 2011 | Female | 79 | NA | NA/NA | NA | Severe AS, moderate AR | | E | | NA |
| Yu and Lee. 2014 | Male | 80 | IV | NA/NA | Multi-organ failure | Severe AS, moderate AR | | A | | Balloon aortovalvuloplasty performed as a bridge  therapy to definite TAVR |
| Bruschi et al. 2014 | Male | 78 | NA | 10.2%/14% | Previous coronary artery bypass surgery and other comorbidities | Severe AS, moderate AR | | A | | NA |
| Sidharta et al. 2015 | Male | 90 | III | 7.57%/8.51% | Chronic obstructive pulmonary disease, pulmonary fibrosis, hypertension, cerebrovascular disease; previous myocardial infarction and PCI to the left circumflex artery with bare metal stent | Severe AS, moderate AR | | B | | NA |
| Ibrahimet et al. 2018 | Female | 82 | NA | NA/NA | Coronary artery disease, hypertension, and peripheral arterial disease | Severe AS, moderate AR | | A | | NA |
| Tohoku et al. 2018 | Female | 85 | NA | NA/NA | Permanent atrial fibrillation | Severe AS, moderate AR | | C | | NA |
| Aoyama et al. 2019 | Male | 83 | NA | NA/NA | Coronary artery disease（rejected surgery） | Severe AS, severe AR | | B | | NA |
| Benkemoun et al. 2020 | Female | 87 | NA | NA/NA | Coronary artery disease（twice post-PCI） | Severe AS | | A | | NA |
| Takahashi et al. 2020 | Female | 84 | NA | NA/NA | NA | Severe AS, severe AR | | A | | Considered non-high-risk of coronary artery occlusion before procedure |
| Fukui et al. 2020 | Female | 74 | NA | NA/7.1% | Higher bleeding risk with recent gastrointestinal bleeding. | Severe AS, moderate AR | | B | | NA |
| Luo et al. 2021 | Male | 62 | III-IV | NA/2 | Symptoms alleviated after medicine therapy | Minor AS, severe AR | | A | | Strong wish to repair aortic valve but refusal of surgical intervention |
| Han et al. 2021 | Male | 70 | NA | NA/NA | NA | AS, AR | | B | | NA |
| Zhou et al. 2021 | Male | 79 | NA | NA/NA | NA | Severe AS, moderate AR | | E | | NA |
|  | Male | 86 | NA | NA/NA | NA | Severe AS, moderate-to-severe AR | | B | | NA |
| Mukherjee et al. 2021 | Female | 75 | III | NA/NA | NA | Severe AS | | B | | NA |
| Liu et al. 2022 | Male | 82 | IV | 18.136%/NA | Hypertension | Severe AR | | A | | Moderate-to-high operative risk |
|  | Male | 72 | IV | 4.055%/NA | Hypertension | Severe AR | | F | |  |
|  | Female | 71 | III | 4.38%/NA | Hypertension | Severe AR | | B | |  |
|  | Male | 69 | III | 2.668%/NA | None | Severe AR | | D | |  |
|  | Male | 75 | III | 8.312%/NA | Heart failure | Severe AS, moderate AR | | B | |  |
| Ochiai et al.2022 | Female | 75 | NA | NA/NA | Diabetes, coronary artery disease, chronic obstructive pulmonary disease | Severe AS, mild AR | | C | | Severe calcified aortic valve and relatively short  membranous septum of 2.3 mm |
| Melania et al. 2022 | Female | 85 | III | NA/NA | Heart failure, coronary artery disease, bicameral pacemaker for grade II Mobitz 2 atrioventricular  blockage | Severe AS, moderate AR | | B | | High-risk for surgery and coronary occlusion with low coronary artery height |
| Zhang et al.2023 | Male | 57 | NA | NA/NA | Acute heart, failure and multiorgan failure, atrial fibrillation | severe AR | | B | | High-risk for surgery and coronary occlusion with low coronary artery height |
| Sato et al. 2023 | Female | 83 | NA | NA/NA | Stanford B type aortic dissection, coronary artery disease, cerebral infarction | Severe AS, moderate AR | | B | | None |
|  | Female | 75 | NA | NA/NA | Coronary artery disease, diabetes mellitus, dyslipidemia, bronchial asthma | | Severe AS, mild  AR | | C | None |
| Aquino-Bruno et al. 2024 | Male | 81 | 3 | 8.10/NA | Hypertension, chronic obstructive pulmonary diseases, frailty | | Severe AS, moderate AR | | B | None |
|  | Male | 79 | 3 | 9.20/NA | Chronic lymphocytic leukemia | | Severe AS, mild  AR | | B | None |
| Bienstock et al. 2024 | Male | 80 | 2.5 | NA/NA | NA | | Severe AS, moderate-to-severe AR | | B | None |

NYHA FC, New York Heart Association functional class; STS, Society of Thoracic Surgeons; EuroSCORE, European system for cardiac operative risk evaluation; QAV, quadricuspid aortic valve; AR, aortic regurgitation; AS, aortic stenosis; PCI, percutaneous coronary intervention; NA, not available.

**Supplementary Table 2. List of all case reports of transcatheter aortic valve implantation in patients with QAV. Preoperative CT measurements.**

| First author and  year of publication | QAV type  (Hurwitz and  Roberts) | Annulus area (mm^2^) | Annulus perimeter (mm) | Sinus diameter(mm) | STJ diameter(mm) | Ascent aorta diameter (mm) | | RCA height(mm) | LCA height(mm) |
| --- | --- | --- | --- | --- | --- | --- | --- | --- | --- |
| Blanke et al. 2011 | E | NA | NA | NA | NA | NA | | NA | NA |
| Yu and Lee. 2014 | A | NA | NA | NA | NA | NA | | NA | NA |
| Bruschi et al. 2014 | A | NA | NA | NA | NA | NA | | NA | NA |
| Sidharta et al. 2015 | B | NA | NA | NA | NA | NA | | NA | NA |
| Ibrahimet et al. 2018 | A | NA | NA | NA | NA | NA | | NA | NA |
| Tohoku et al. 2018 | C | 372 | NA | NA | NA | NA | | NA | NA |
| Aoyama et al. 2019 | B | 483.7 | 79.1 | NA | NA | NA | | 16.1 | 11.8 |
| Benkemoun et al. 2020 | A | NA | NA | NA | NA | NA | | NA | NA |
| Takahashi et al. 2020 | A | NA | NA | NA | NA | NA | | NA | NA |
| Fukui et al. 2020 | B | NA | NA | NA | NA | NA | | NA | NA |
| Luo et al. 2021 | A | NA | NA | NA | NA | NA | | NA | NA |
| Han et al. 2021 | B | 527.7 | 83.3 | NA | NA | NA | | NA | NA |
| Zhou et al. 2021 | E | NA | NA | NA | NA | NA | | NA | NA |
|  | B | NA | NA | NA | NA | NA | | NA | NA |
| Mukherjee et al. 2021 | B | NA | NA | NA | NA | NA | | NA | NA |
| Liu et al. 2022 | A | 555.7 | 84.3 | LC: 33.1mm, RC: 36.6mm | 30.3 | NA | | 18 | 14.1 |
|  | F | 635.5 | 90.3 | LC: 33.6mm, RC: 34.9mm | 29.5 | NA | 13.6 | | 13.3 |
|  | B | 500.5 | 80.2 | LC: 38.2mm, RC: 35.9mm | 28.9 | NA | | 6.9 | 7.5 |
|  | D | 680.4 | 93.3 | LC: 39.1mm, RC: 40.7mm | 33 | NA | | 18.5 | 8.7 |
|  | B | 605.3 | 87.5 | LC: 37.9mm, RC: 40.7mm | 26.9 | NA | | 12.3 | 9.7 |
| Ochiai et al.2022 | C | 363.5 | 68.3 | NA | NA | NA | | NA | NA |
| Melania et al. 2022 | B | NA | NA | NA | NA | NA | | NA | NA |
| Zhang et al.2023 | B | 544 | 84.1 | NA | 34.7 | 42 | | 8.9 | 12.8 |
| Sato et al. 2023 | B | NA | NA | NA | NA | NA | | NA | NA |
|  | C | NA | NA | NA | NA | NA | | NA | NA |
| Aquino-Bruno et al. 2024 | B | 369.7 | 69.7 | NA | 25.8 | NA | | 16.5 | 15.8 |
|  | B | 325.4 | 65.9 | NA | 23.3 | NA | | 12.4 | 11.5 |
| Bienstock et al. 2024 | B | NA | NA | NA | NA | NA | | NA | NA |

QAV, quadricuspid aortic valve; LC, left coronary; LCA, left coronary artery; RC, right coronary; RCA, right coronary artery; STJ, sinotubular junction.

**Supplementary Table 3. List of all case reports of transcatheter aortic valve implantation in patients with QAV. Periprocedural and Follow-up data.**

| First author and  year of publication | TVAR device used | Valve size (mm) | Approach | Procedural outcomes | Procedural complications | In-hospital and 30-day  complication | Follow-up duration | Follow-up performance | Otherwise Specified |
| --- | --- | --- | --- | --- | --- | --- | --- | --- | --- |
| Blanke et al. 2011 | Edwards Sapien | 26 | TAp | Success | None | NA | NA | NA | None |
| Yu and Lee. 2014 | Edwards Sapien XT | 26 | TF | Success | None | NA | NA | NA | None |
| Bruschi et al. 2014 | Medtronic CoreValve | 29 | TF | Success | Trivial PVL | NA | NA | NA | None |
| Sidharta et al. 2015 | PORTICO THV (a self-expanding,  and repositionable valve) | 27 | TF | Success | Minor PVL | None | 1 month | Significant symptom improvement (NYHA II) | The first documented case of TAVR with a self-  expandable, fully repositionable and recapturable valve for the treatment of stenosed QAV. |
| Ibrahimet et al. 2018 | Edwards Sapien 3 | 23 | TF | Success | None | None | NA | NA | None |
| Tohoku et al. 2018 | Edwards SAPIEN 3 | 23 | TF | Success | Trivial PVL | None | NA | NA | None |
| Aoyama et al. 2019 | Evolut R | 29 | TF | Success | None | None | NA | NA | None |
| Benkemoun et al. 2020 | Edwards SAPIEN 3 | 23 | TF | Success | None | None | 6 months | None | None |
| Takahashi et al. 2020 | Edwards SAPIEN 3 | 23 | TF | Success | Hemodynamically unstable just after the prosthetic valve was deployed. Found 99% stenosis of the left main coronary artery acquired reperfusion and stable hemodynamics after a stent deployed. | NA | NA | NA | None |
| Fukui et al. 2020 | Edwards SAPIEN 3 | 23 | TF | Success | None | None | 3 months | None | None |
| Luo et al. 2021 | J-Valve | 27 | TAp | Success | None | None | 6 months | None | None |
| Han et al. 2021 | VenusA | 26 | TF | The first valve with severe PVL， the second one (implanted  10mm  higher) with moderate PVL | Moderate PVL | NA | NA | NA | None |
| Zhou et al. 2021 | VenusA | 23 | TF | Success | None | None | 5 years | None | None |
|  | Edwards SAPIEN XT | 26 | TF | Success | None | None | 3 years | None | None |
| Mukherjee et al. 2021 | Edwards SAPIEN 3 | 23 | TF | Success | A coronary stent parked for protection | None | 1 month | None | RCA occlusion by displaced leaflets after predilation |
| Liu et al. 2022 | J-Valve | 27 | TAp | Success | Bleeding and transfusion needed | None | Median follow-up period: 18 (12–56) months | 1-year LVEF：51%，NYHA III | None |
|  | J-Valve | 29 | TAp | Success | None | None |  | 1-year LVEF：48%，NYHA II | None |
|  | J-Valve | 27 | TAp | Success | None | None |  | 1-year LVEF：51%，NYHA I | None |
|  | J-Valve | 29 | TAp | Success | Trival PVL | PPI for advanced A-V block |  | 1-year LVEF：54%，NYHA I | None |
|  | VenusA | 32 | TF | Success | None | None |  | 1-year LVEF：45%，NYHA II | None |
| Ochiai et al.2022 | Edwards SAPIEN 3 | 23 | TF | Success | None | None | NA | None | None |
| Melania et al. 2022 | A balloon-expandable valve | NA | NA | Success | Coronary protection and a chimney  stenting performed after implantation | None | None | NA | NA |
| Zhang et al.2023 | J-Valve | 29 | TAp | Success | Minor PVL | None | 6 months | None | None |
| Sato et al. 2023 | Edwards Sapien 3 | 23 | TF | Success | None | None | 4 years | None | None |
|  | Edwards Sapien 3 | 23 | TF | Success | None | None | NA | NA | None |
| Aquino-Bruno et al. 2024 | Edwards Sapien 3 | 23 | TF | Success | None | None | 6 months | None | None |
|  | Edwards Sapien 3 | 23 | TF | Success | None | None | 6 months | None | None |
| Bienstock et al. 2024 | Evolut FX | 26 | TF | Success | None | NA | None | NA | None |

TF, transfemoral; TAp, transapical; LVEF, left ventricular ejection fraction; PVL, paravalvular leak; TAVR, transcatheter aortic valve replacement; PCI, percutaneous coronary intervention; NA, not available.
